# LDL-cholesterol measurement in diabetic type 2 patients: a comparison between direct assay and popular equations

**DOI:** 10.1186/s40200-017-0326-2

**Published:** 2017-11-03

**Authors:** Farideh Razi, Katayoon Forouzanfar, Fatemeh Bandarian, Ensieh Nasli-Esfahani

**Affiliations:** 1 Diabetes Research Center, Endocrinology and Metabolism Clinical Sciences Institute, Tehran University of Medical Sciences, North Kargar, Tehran, 1411413137 Iran; 2 Endocrinology and Metabolism Research Center, Endocrinology and Metabolism Clinical Sciences Institute, Tehran University of Medical Sciences, Tehran, Iran

**Keywords:** Diabetes, LDL-cholesterol, Friedwald formula

## Abstract

**Background:**

Low density lipoprotein –Cholesterol (LDL-C) is one of the main factors for assessment of cardiovascular disease risk and it is more important in diabetic patients. Various methods are currently used for LDL-C measurements which are compared in this study.

**Methods:**

This study was conducted in Diabetes Research Center based on laboratory results of 1721 diabetic patients who referred to laboratory for regular follow-up of lipid profile. LDL-C was measured directly and also estimated according to Friedwald, Anandraja and Chen formulas.

**Results:**

Results of direct LDL-C measurements were lower than all calculations at triglycerides (TG) levels less than 150 mg/dL while in higher TG levels direct measurement values were higher than Friedwald and Anandraja formula. Friedwald and Chen formula results had better correlation(r) with direct measurement than Anandraja in different levels of TG and also were able to define LDL-C > 100 mg/dL more accurately.

**Conclusions:**

Although we observed excellent correlation between the studied formulas with direct measurement, using the formula can misclassified diabetic patients with LDL-C values near threshold (100 mg/dL). However calculated LDL-C based on Chen and Friedwald formula can be a suitable alternative for direct measurement especially in regions with limited resources**.**

## Background

Diabetes mellitus is a metabolic disease, which contributes to premature mortality worldwide [1, 2]. The number of diabetic people is increasing due to population growth and high prevalence of physical inactivity and obesity. The global prevalence of diabetes in adults is estimated to increase from 8.8% in 2015 to 10.4% in 2040 [3] as a major health problem. Different studies have been conducted to depict the figure of diabetes and its complications [4–7] as well as its management status [8]. Cardiovascular diseases (CVD) are among the main comorbid conditions associated with diabetes, which causes about 70% of deaths in diabetics aged more than 65 years [9]. Due to this high rate of mortality, CVD risk score should be determined in diabetic patients for prevention and further management [10]. Dyslipidemia is another common comorbid disorder of diabetes, which increase the risk of CVD in diabetic patients [6]. One of the main factors used for CVD risk assessment is serum level of low density lipoprotein –Cholesterol (LDL-C) and it is important to keep it less than 100 mg/dL in diabetic patients [11].

Different methods are used for measuring serum LDL-C concentration. Ultracentrifugation following by beta-quantification is the gold standard for measurement of LDL-C [12] but special equipment requirement, expensiveness and time-consuming of this method make it inconvenient for most routine clinical laboratories [13]. So other methods such as homogenous assay techniques are widely used for direct measurement of LDL-C as well as different formulas such as Friedewald, Chen and Anandraj [14–16].

In order to manage diabetic patients, it’s very important to use suitable laboratory methods and achieve accurate results especially at decision level for essential parameters used for follow-up. Since many laboratories usually use formula to report LDL-C for patients (including diabetic patients) it is necessary to know about the agreement of results obtained by these different methods. This study’s aim was to compare these formulas and direct measurement method in a large sample of diabetic patients to find the most accurate and reliable method for measuring serum LDL-C.

## Material and methods

In this study, all laboratory results of patients with type 2 diabetes admitted to Diabetes Research Center (affiliated to Endocrinology and Metabolism Clinical Sciences Institute) from March to July 2016, were evaluated retrospectively and lipid profile results were extracted. The laboratory method for measurement of LDL-C, high density- cholesterol (HDL-C), total cholesterol (TC), and triglycerides (TG) was enzymatic photometric method [17] using commercial kits (Pars Azmun, Iran) and biochemical autoanalyser (Prestige 24i, Tokyo Boeki Medical System, Japan). Total precision according to coefficient variation (CV %) for measuring TC, TG, HDL-C and LDL-C was 1.6, 1.5, 2.4 and 2.3, respectively.

In the next step LDL-C was calculated by three following formulas:

Friedwald Equation: LDL = TC – HDL − (TG / 5).

Anandraja Equation: LDL = (0.9 TC) − (0.9 TG/5) −28.

Chen Equation: LDL = (TC − HDL) × 0.9 − (TG × 0.1).

The calculated LDL-C values were compared to the results of direct measurement of LDL-C (all the values are expressed in mg/dL).

### Statistical analyses

Data analyses were performed using SPSS software ver. 21.00 for Windows (SPSS Inc., Chicago, IL). Data were classified according to TG level into six groups and in each group correlation of formulas and directly measured LDL-C values, were tested by Pearson’s correlation. Wilcoxon test was also used to estimate the differences between groups. Regression curve and Bland–Altman plots were used to evaluate the agreement and absolute difference between the three formulas and the directly measured LDL-C, respectively. A *P* value less than 0.05 was considered statistically significant.

In the next step LDL-C results obtained from direct measurement were categorized into two groups considering 100 mg/dL as cut off point and, sensitivity and specificity of each formula for identification of LDL-C ≥ 100 mg/dL was estimated.

## Results

We evaluated the results of lipid profile of 1721 patients with type 2 diabetes. The number of female and male patients were 874(50.8%) and 847(49.2%), respectively. Table 1 shows distribution of age and lipid profile as well as calculated LDL-C values in both genders. The mean serum TC, HDL-C and LDL-C were significantly higher in female patients.

**Table 1:** Age, lipid profile and calculated LDL-C results in diabetic patients and differences according to sex

Variable	Sex	Mean	SD	*P*-value
Age (year)	F	58.1	10.1	0.1
	M	58.9	11.2	
TG (mg/dL)	F	154.3	81.7	0.4
	M	150.9	91.5	
TC (mg/dL)	F	168.9	38.5	<0.001
	M	154.9	40.5	
HDL-C(mg/dL)	F	48.1	10.8	<0.001
	M	41.1	8.8	
LDL-C, Direct method (mg/dL)	F	88.1	24.4	<0.001
	M	82.4	24.7	
LDL-C,Friedwald (mg/dL)	F	89.9	31.3	<0.001
	M	83.6	31.9	
LDL-C,Anandraja (mg/dL)	F	96.3	32.0	<0.001
	M	84.3	31.6	
LDL-C,Chen (mg/dL)	F	93.4	29.5	<0.001
	M	87.4	30.5	

F: female. M: male, TG: Triglyceride, TC: Total cholesterol, LDL-C: low density lipoprotein-cholesterol, HDL-C: high density lipoprotein-cholesterol

Table 2 shows mean and SD of LDL-C values in all groups (direct assay, Friedwald, Anandraja and Chen) classified according to TG level. Results of direct measurement of LDL-C were lower than all calculations in the group of patients with TG level less than 150 mg/dL. While in higher TG levels, direct measurement values were higher than Friedwald and Anandraja formula. Friedwald and Chen formula results had better correlation(r) with direct measurement than Anandraja in different levels of TG. All differences were significant in TG < 400 mg/dL.

**Table 2:** Differences between direct measurement of LDL-C and calculation, sensitivity and specificity (using an LDL-C cut-off of 100 mg/dL) in different TG levels

TG	LDL-C	*N*	Mean(SD)	Z (*P*-value)	r (*P*-value)	Sensitivity	Specificity
<50(mg/dL)	Direct (mg/dL)	41	66.4 (14.5)				
	Friedwald(mg/dL)	41	73.2(20.1)	−4.5 (<0.001)	0.96 (<0.001)	100.0	92.5
	Anandraja(mg/dL)	41	86.4 (24.8)	−5.5 (<0.001)	0.86 (<0.001)	100.0	77.5
	Chen(mg/dL)	41	69.4 (18.2)	−3.0 (0.003)	0.96 (0.002)	100.0	97.5
51–150 (mg/dL)	Direct (mg/dL)	983	79.3(21.3)				
	Friedwald(mg/dL)	983	84.3 (28.2)	−16.1 (<0.001)	0.98 (<0.001)	100.0	89.3
	Anandraja(mg/dL)	983	89.9 (29.5)	−21.8 (<0.001)	0.94 (<0.001)	98.7	80.7
	Chen(mg/dL)	983	84.2 (25.6)	−20.7 (<0.001)	0.98 (<0.001)	100.0	91.3
151–300 (mg/dL)	Direct (mg/dL)	597	92.7(25.3)				
	Friedwald(mg/dL)	597	90.6(34.6)	−5.2 (<0.001)	0.98 (<0.001)	90.0	94.8
	Anandraja(mg/dL)	597	91.2 (34.9)	−35.0 (<0.001)	0.96 (<0.001)	92.4	92.2
	Chen(mg/dL)	597	97.7 (31.5)	−13.6 (<0.001)	0.99 (<0.001)	99.0	88.4
301–400 (mg/dL)	Direct (mg/dL)	70	104.1(27.6)				
	Friedwald(mg/dL)	70	94.0 (41.3)	−4.7 (<0.001)	0.98 (<0.001)	83.3	100.0
	Anandraja(mg/dL)	70	90.4 (42.5)	−5.6 (<0.001)	0.98 (<0.001)	65.0	100.0
	Chen(mg/dL)	70	111.9 (37.1)	−4.9 (<0.001)	0.95 (<0.001)	100.0	91.2
401–500 (mg/dL)	Direct (mg/dL)	21	118.6 (31.3)				
	Friedwald(mg/dL)	21	107.8(49.8)	−1.9 (<0.001)	0.99 (<0.001)	92.9	100.0
	Anandraja(mg/dL)	21	104.9(50.8)	−2.3 (0.02)	0.98 (0.007)	92.9	100.0
	Chen(mg/dL)	21	131.6(45.1)	−2.9 (<0.003)	0.99 (<0.001)	100.0	100.0
501–1000 (mg/dL)	Direct (mg/dL)	9	113.4(26.8)				
	Friedwald(mg/dL)	9	72. 9(50.1)	−2.0 (0.038)	0.51 (0.02)	28.6	100.0
	Anandraja(mg/dL)	9	69.9(46.2)	−2.4 (0.02)	0.58 (<0.008)	28.6	100.0
	Chen(mg/dL)	9	121.0(44.2)	−0.59 (0.55)	0.64 (<0.50)	71.4	100.0

Table 2 also shows that Chen and Friedwald formula had high sensitivity and specificity when TG levels were less than 500 mg/dL. Surprisingly LDL-C calculated by Chen formula had acceptable sensitivity and specificity in group with TG: 400–500 mg/dL.

**Fig. 1:**
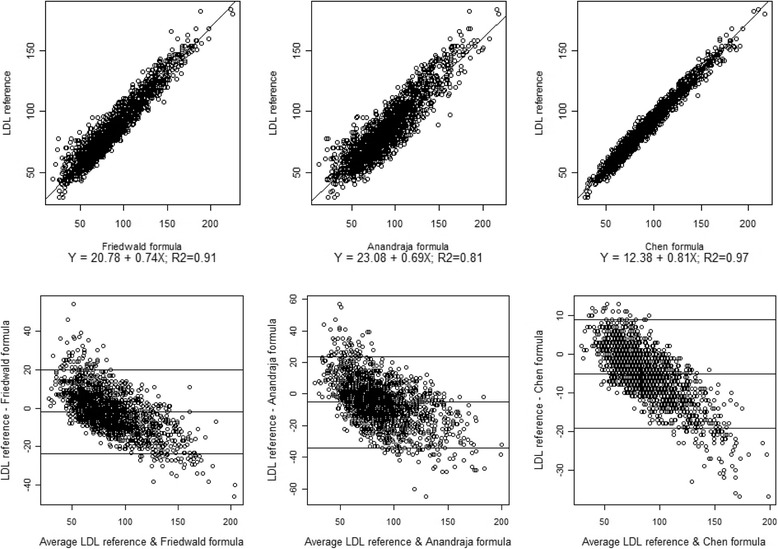
Upper row: linear regression curve and formula. Chen formula results have better correlation with direct assay results. Lower row: Bland–Altman difference plots of the directly measured LDL-C and the LDL-C derived from the three formulae. The mean bias for each calculation were: Friedewald formula −1.73 ± 11.08, Anandraja formula: −5.21 ± 14.68 and the Chen formula: −5.12 ± 7.17 mg/dL

Figure 1 shows regression plot and also Bland Altman difference plot between direct measurement and each formula. Results obtained by formulas were correlated with direct measurement but in higher LDL-C values, results obtained by formula were higher than direct measurement while in lower LDL-C values the results were lower than direct assay values.

## Discussion

The results of this study showed that calculated LDL-C values are higher than direct measurement in TG < 150 mg/dL while in TG > 150 mg/dL the results are inverse (except Chen formula results which were higher in the whole range of TG). LDL-C values obtained by all formulas had excellent correlation with direct assay results in TG concentration less than 500 mg/dL but the results were significantly different. In mentioned level of TG, formulas gave results with good sensitivity and specificity to detect LDL-C more than 100 mg/dL. But in TG > 500 mg/dL correlation of all formulas’ results with direct measurement was poor.

The Friedewald formula, is based on this theoretical principle that the ratio of TG to cholesterol in very low density lipoprotein (VLDL) is fairly stable (5:1) and validated in a group of normal subject and also primary hyperlipoproteinemia (Type II and IV) [18]. This formula is widely used by clinical laboratories and also recommended by National Cholesterol Education Program (NCEP) [19]. Friedwald formula has been evaluated in different studies. By examination of more than ten thousand individual in Brazil, De Cordova [20] showed that Friedwald formula has positive bias at TG < 150 mg/dL, no bias at TG 150–300 mg/dL and negative bias at TG level 300-400 mg/dL, which their results are very similar to our study. They suggested a new formula for LDL-C calculation: LDL = 0.75 (TC − HDL), which is not dependent on TG level [21]. Some studies on general population and diabetic patients have shown that Friredwald formula give significantly higher results compared to direct method [14]. In a study on diabetic patients same result was obtained [22] but in two separate studies on diabetic patient, Friedewald equation showed negative bias which this issue can be very important in values near the cutoff points [23, 24]. According to American Diabetes Association (ADA) guideline in diabetic patient without overt CVD, the goal of serum LDL-level is < 100 mg/dL [25]. By using 100 mg/dL as the threshold level, more than 10% of patients will be misclassified, if Friedwald formula compared to direct measurement of LDL-C is used, this is the same as results reported by Chai Kheng et al. [24].

Other researchers have suggested new formula to improve calculated LDL-C results such as Anandraja [15] and Chen [16]. Although these formulas are not conventional, they have been investigated in this research. In our study the results of Anandraja formula were fairly correlated with direct measurement in most TG level categories. The results of Chen formula were more comparable to direct measurement even better than Friedwald results as in the original article [16] and Martin’s study [26].

Quality of methods for direct measurement of LDL-C compared to the reference measurement procedure has been evaluated in various studies [12, 27] and found to be comparable to the reference method. But direct assays for LDL-C measurement are expensive and there is necessity to find an alternative method to decrease the cost of laboratory methods. The results of present study might have some limitations due to lack of reference method for LDL measurement.

## Conclusion

Although we observed excellent correlations between the evaluated formulas and direct measurement, using the formula can misclassified patients with LDL-C values near threshold (100 mg/dL). However calculation of LDL-C based on Friedwald and Chen formula can be a good alternative for direct measurement especially in regions with limited resources**.**
